# Evidence-Based Medicine: History, Review, Criticisms, and Pitfalls

**DOI:** 10.7759/cureus.35266

**Published:** 2023-02-21

**Authors:** Iqbal Ratnani, Sahar Fatima, Muhammad Mohsin Abid, Zehra Surani, Salim Surani

**Affiliations:** 1 Center for Critical Care, Houston Methodist Hospital, Houston, USA; 2 Internal Medicine, Greater Baltimore Medical Center, Baltimore, USA; 3 Medical Education and Simulation, It's Your Life Foundation, Corpus Christi, USA; 4 Anesthesiology, Mayo Clinic, Rochester, USA; 5 Medicine, Texas A&M University, Corpus Christi, USA; 6 Medicine, University of North Texas, Dallas, USA; 7 Internal Medicine, Pulmonary Associates, Corpus Christi, USA; 8 Clinical Medicine, University of Houston, Houston, USA

**Keywords:** medical education technology, pitfalls of ebm, constructive criticism, history of ebm, evidence base medicine

## Abstract

Evidence-based medicine (EBM) is the use of high-quality clinical research in making decisions about the care of patients. Its formal origin dates back to the mid-nineteenth century, and since then, it has continued to evolve. The best research evidence, clinical expertise, and patient values are described as the foundations of EBM. However, several tools and skills have been developed and added over time. EBM has faced a lot of criticism, and the pitfalls are widely discussed and published in the medical literature. The biggest challenge is the changing paradigm of healthcare, cost-effectiveness, and changing evidence which has led to controversies and challenges in the rapid adaptation of the EBM. This review article discusses the history, conception, and evolution of modern-day EBM. In addition, we discuss why EBM has been criticized and highlight the pitfalls.

## Introduction and background

A brief history of EBM 

Although it has been claimed that the philosophical origins of EBM extend back to the mid-19th century in Paris, France, or even earlier, the term "evidence-based medicine" (EBM) first appeared in the 1991 American College of Physicians (ACP) Journal Club editorial [1-2]. The roots of modern EBM go back approximately 50-60 years, with similar works emerging at various institutions in North America and Europe [1-2].

Alvan Feinstein was a mathematician turned physician. While working on rheumatic fever in a hospital in New York during the mid-1950s, he realized there were no clinical criteria to distinguish between benign and pathological murmurs. Instead, it solely depended on the physician's experience. He classified the murmur on scientific grounds, and his work improved the outcome [3].

Dr. David Sackett of McMaster University formalized the EBM movement in 1967. Clearly, it expressed his vision as "the application, by a physician who provides direct patient care, of epidemiological and biometric methods to the study of the diagnostic and therapeutic process to effect a health improvement" [4]. In 1981, Dr. David Sackett and his colleagues introduced the term "critical appraisal," a method for understanding literature and its application at the bedside [5]. His student, Guyatt et al., continued to expand upon his work and, a decade later, led to the formation of an international EBM working group (Evidence-Based Medicine Working Group, 1992). The work of this group resulted in the birth of the famous "User's Guide to the Medical Literature," which transformed from a series of articles into a periodically updated textbook [6].

The history of EBM would be incomplete without the mention of Suzanne and Robert Fletcher, scholars at the Robert Wood Johnson Clinical Scholars Program. They were among the first to describe the absence of the translational application of biomedical science to clinical medicine. After decades of work, they eventually published their work describing the scientific basis of epidemiology for clinical care in a textbook format named Clinical Epidemiology: The Essentials [7]. Though the Cochrane Collaboration is only 29 years old, its history traces back to World War (WW) II. It is named after the Scottish physician Archibald (Archie) Cochrane, himself a victim of porphyria. He was an ardent believer in randomized control trials (RCTs). His works include the effect of yeast on unexplained generalized edema in Prisoners of War (POW) [8-9]. Later, he became a pioneer in the epidemiology of tuberculosis and was the first to report the correlation between cigarette smoking and lung cancer [8-9].

Daly reported in her book Evidence-Based Medicine and the Search for a Science of Clinical Care that Lian Chalmers, an English obstetrician and epidemiologist, learned his lesson about the dangers of not using antibiotics early in malnourished children while working for two years (1969-70) in Palestinian refugee camps in Gaza [10]. Lian Chalmers joined hands with Thomas Chalmers, a physician and another advocate of RCTs and meta-analyses, who was interested in advancing the work of Archie Cochrane [11].

The Cochrane Collaboration has grown tremendously over the last 29 years. They have representation from over 130 countries in the form of more than 13,000 members and 87,000 supporters [11]. Besides the massive library of a gigantic database of trials, systematic reviews, meta-analyses, guidelines, and evidence-based resources, it is also a source of learning on conducting, editing, and reading systematic reviews [12].

This evidence-based approach worked well for most of the patients; however, this one-size-fits-all approach does not consider the differences between each patient. Each human is different and is a complex form of various biological systems. There may be a significant subgroup of patients who have not responded to or benefited from the said processes and are subject to a bias of statistical insignificance [13]. A substantial number of "human beings" may be outliers and suffer from inferior medical treatment as they may fall away from the median or mean of the bell curve. This led many researchers to question the validity of research evidence [14]. This review is an attempt to bring to light the research question: How viable and applicable is the practice of Evidence-Based Medicine for each patient at the personal level?

## Review

EBM has been at the forefront of medicine for improving the quality of care and cost-effective medical care. These are based on dimensions, foundations, and principles. We will explore these further.

Dimensions

**Figure 1:**
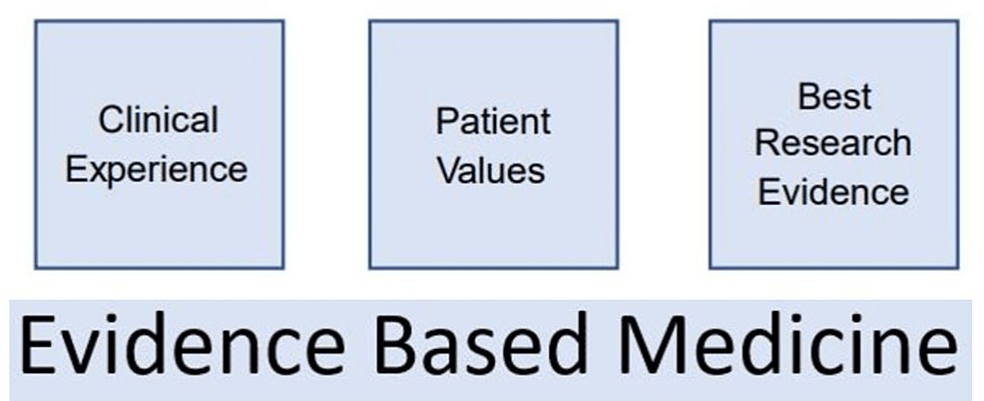
Three original dimensions of EBM (picture is created by the authors of this article) The three original dimensions though include clinical expertise and patient values, over time more emphasis led only on research evidences.

The three originally described philosophical dimensions of EBM were (a) the best research evidence, (b) clinical experience, and (c) patient values, as shown in Figure 1. This has been the fundamental basis of the EBM. These three dimensions help to cover the areas of research, clinical, and patient care and help us lead toward patient-centered care based on the best research and clinical expertise [15-16].

Foundations

**Figure 2:**
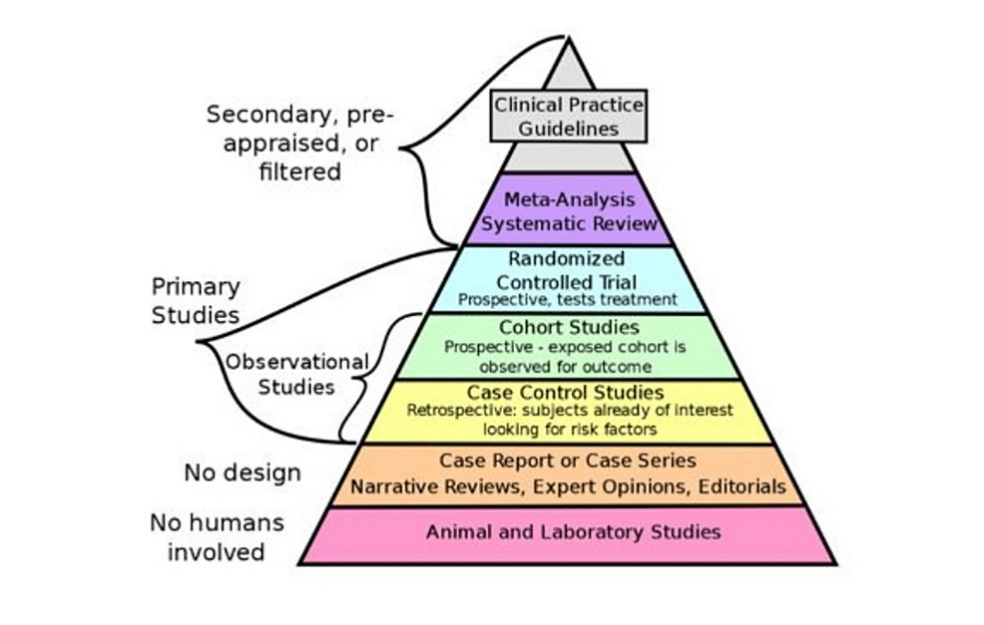
A typical pyramid of evidence in EBM (Copyright: free to use under Creative Commons Attribution-Share Alike 4.0 International license) A typical pyramidal journey of medical science research work from animal and laboratory studies to the development of clinical guidelines [17].

The foundation of EBM rests on clinical studies. Various pyramids have been developed depending on the strength of different clinical studies. Five levels of evidence described by Sackett still serve as the basis for most guidelines, though variations and modifications have been adopted, as shown in Figure 2 [15-16]. Systematic reviews of RCTs and robust RCTs still serve as the necessary foundations for EBM. The five levels of evidence are: (i) level I large RCTs with precise results; (ii) level II small RCTs with unclear results; (iii) level III cohort and case-control studies; (iv) level IV historical cohort or case-control studies; and (v) level V case series studies with no controls.

Principles

As described above, the "Cochrane" database is the most developed, organized, and multi-national institution for implementing EBM in healthcare, with solid roots in history and philosophy. Since "Cochrane" has incorporated training, it has changed its original name from "Cochrane collaboration" to simply "Cochrane" and has devised ten principles (Table 1) [18].

**Table 1:** "Cochrane" ten principles "Cochrane" database is considered the most developed, organized, and multi-national institution for implementing EBM in healthcare.

Principles	Application
Collaboration	By fostering global cooperation, teamwork, and open and transparent communication and decision-making.
Building on the enthusiasm of individuals	By involving, supporting, and training people of different skills and backgrounds.
Avoiding duplication of effort	By good management, coordination, and effective internal communications maximize the economy of effort.
Minimizing bias	Through a variety of approaches such as scientific rigor, ensuring broad participation, and avoiding conflicts of interest.
Keeping up to date	By a commitment to ensure that Cochrane Reviews are maintained through the identification and incorporation of new evidence.
Striving for relevance	By promoting the assessment of health questions using outcomes that matter to people making choices in health and health care.
Promoting access	By wide dissemination of our outputs, taking advantage of strategic alliances, and promoting appropriate access models and delivery solutions to meet the needs of users worldwide.
Ensuring quality	By applying advances in methodology, developing systems for quality improvement, and being open and responsive to criticism.
Continuity	By ensuring that responsibility for reviews, editorial processes, and key functions is maintained and renewed.
Enabling Wide Participation	In our work by reducing barriers to contributing and encouraging diversity.

The Center for Evidence-Based Management, based in the Netherlands, has developed a concise set of six principles of EBM, known as the 6A's (Table 2), and a skill tool called PICOC (population, intervention, comparison, outcome, and context) (Table 3) [19-20].

**Table 2:** Six A's of CEBMA The Center for Evidence-Based Management (CEBMA) proposed 6 A's.

Asking	Translating a practical issue or problem into an answerable question.
Acquiring	Systematically searching for and retrieving the evidence.
Appraising	Critically judging the trustworthiness and relevance of the evidence.
Aggregating	Weighing and pulling together the evidence.
Applying	Incorporating the evidence into the decision-making process.
Assessing	Evaluating the outcome of the decision taken.

**Table 3:** Skill tool PICOC The Center for Evidence-Based Management (CEBMA) proposed a skill tool called PICOC (population, intervention, comparison, outcome, and context).

Population	Who?
Intervention	What or how?
Comparison	Compared to what?
Outcome	What are you trying to accomplish? (objective)
Context	In what kind of organization/circumstances?

Criticism and pitfalls of EBM 

EBMs have significant criticism and drawbacks. Critics have been cautioning about the outfalls of EBM since its inception. Interestingly, Alvan Feinstein, who was described in the history of EBM as one of the earliest pioneers of clinical epidemiology, criticized EBM more than two decades ago in the following words: "The laudable goal of making clinical decisions based on evidence can be impaired by the restricted quality and scope of what is collected as "best available evidence." However, the authoritative aura given to the collection may lead to significant abuses that produce inappropriate guidelines or doctrinaire dogmas for clinical practice [20]. He believed that the foundations of EBM are based on statistical values, and statistics textbooks overlook statistical heterogeneity [21].

Curse of the 'p-Value'

All RCTs are eventually judged by the magic number of a p-value <0.05. For a long time, statisticians have been warning against overzealous reliance on the p-value. In harsh words, a term called "p-hacking" has been invented [22]. The misuse of p-value may lead to highly confounded and biased studies between two comparative groups as it may overzealously rely on sub-groups or vice versa. Data can be manipulated to obtain p-values. This leads some authors to take an extreme stand against all studies [23].

Absence of N of 1 Trial 

N of one trial is considered the best way of establishing causality. As the name implies, a single patient is an entire trial. A random allocation of an experimental and a control intervention are given to a patient. It is an RCT of one patient. This is, in one way, a targeted experiment but very hard to carry out regularly [24].

The Problem of Validities Within Systematic Reviews and Meta-Analyses

All systemic reviews are dependent on a collection of studies. This creates a problem as patient selection criteria differ in different studies, bringing into question the internal and external validity of any systematic reviews [25].

Publication bias: Popularly known as "grey literature," unpublished studies are an enigma for researchers. Many systematic reviews do not include unpublished studies [26]. This may be due to their inherent problems of the likelihood of poor quality (which may be biased), difficulty locating, and the unlikelihood of being peer-reviewed.

*Clinical Expertise and Patient Values are Increasing Being Sidelined* 

It has been proposed that the two primary components of the initially proposed EBM appear to be more and more sidelined in clinical practice with protocolized, algorithm, and guideline-based treatments. Clinicians have little room for judgment, and patients have little respect for their choices. Medicine is becoming more of a managerialism of templates, and there is a creeping politicization of clinical practice [27]. The term "problem of extra information" (PEI) has been devised to argue that physicians' value of judgment - a piece of extra information - plays an integral role in managing any disease. EBM takes away that crucial part of clinical medicine [28].

Reimbursements and Incentives Tied to Guidelines 

More and more reimbursements and incentives by the government and insurance companies are becoming tied to the practice of medicine according to approved guidelines and protocols. Physicians are being constrained in their clinical judgment. The same is true for patient choices. Clinicians are worried that they are increasingly relying on others to synthesize the science, and consensus-based guideline panels are moving them back toward authority-based medicine [29].

The Pileup of Never-Ending Guidelines and EHR Compliance

Since guidelines have become the backbone of EBM and reimbursement, medical societies in almost all sub-specialties have started putting out never-ending policies. Several years ago, an audit of a 24-hour medical intake in an acute hospital, including 18 patients with 44 diagnoses, identified 3679 pages of national guidelines (an estimated 122 hours of reading) relevant to their immediate care [30]. The situation seems to worsen as Electronic Health Records (EHR) are now mandatory in the United States of America (USA). All hospitals integrate guidelines, protocols, pre-populated orders, and checklists for physicians to comply with. Physicians spend more time on the EHR than face-to-face with patients [31].

EBM is a Poor Fit in Multi-Morbid Situations and Hinders' Systems Thinking'

Most of the RCTs, systematic reviews, and meta-analyses, which are the backbone of EBM, and guidelines are unimodal, which means they do not consider the aging population, who may have a cluster of many morbid diseases [32]. Clinical expertise is needed to create a well-crafted plan for each patient, but EBM fails to provide clinicians with much room for such activity. Clinicians are heavily burned out. They are short on time to justify or document any omission or deviation from the required parameters. One unintended fallout of such failure is polypharmacy and its dangers [33]. This unimodal approach goes against the holistic approach of "systems thinking" required in patients' care.

Physicians' Ignorance of Statistics

Statistics play an integral role in the interpretation of EBM. Physicians have minimal knowledge of interpreting and critically examining RCTs due to their minimal statistical knowledge. This results in the acceptance of guidelines without any critical evaluation [34].

*Ignoring 'Weak Signals' and 'Long Tail'* 

Many clear signs of progress have been made by paying attention to the 'weak signals'. They can be described as sporadic case reports/series without substantial backup from robust trials in the medical literature. They may be the signals of 'red flags' for future events or incoming catastrophes [35]. EBM, at its core, relies on the "bell curve" of RCTs. But there is an increasing realization that the 'long tail' may have enormous strength. Considering the bell curve as the sole power source would be a mistake [36].

New Organizations

In recent years, many for-profit and non-profit organizations have been formed. The most famous is the Center for Evidence-Based Medicine Management [37], the University of Oxford's Center for Evidence-Based Medicine [38], the Patient-Centered Outcomes Research Institute (PCORI) [39], and others, all striving for the exact cause. Efforts are being undertaken to educate on critically reviewing the literature and RCT, but it lags behind in medical student and residency programs. When entering the practice, physicians and allied health practitioners rely on pharmaceutical companies and societies that may have their own biases [40].

Recent Thoughts

A very recent article published less than a year ago in the British Medical Journal claimed that over time the practice of EBM has been corrupted due to corporate interests, failed regulation, and the commercialization of academia [41]. There is a need for evolution rather than an adaptation of EBM due to the introduction of artificial intelligence (AI) and technology in medicine. To summarize the research question asked at the beginning of this article, there is a need for a merger of EBM towards personalized medicine through methodological advances and future AI-based data analyses of all data to offer the right treatment to the right patient at the right time [42].

## Conclusions

Health professionals have used EBM to guide clinical decision-making for the last three decades. However, critics have suggested that EBM focuses on groups of patients and does not consider the differences between each patient, subgroup analyses, or patient values and preferences. There is an emergent need to address the gaps at various levels to synchronize the functions of different paradigms of the practice of EBM. The gaps created in the practice of EBM by the evolving technology, integration of data science, and integration of AI in medicine are consequently impacting the physicians' learning in training. As a result of the Domino effect, there is an evaporating trust in the healthcare system. This gap can be addressed by integrating new scientific paradigms to facilitate the learning of the next generation of physicians.
